# The Challenges of New Endovascular Technologies

**DOI:** 10.1186/s42155-019-0086-9

**Published:** 2019-12-12

**Authors:** Jim A. Reekers

**Affiliations:** Professor Emeritus Interventional Radiology, Amsterdam UMC University of Amsterdam, Amsterdam, Netherlands

At the last Veith meeting in New York, I was overwhelmed by the enormous amount and variety of endovascular tricks and especially all the new endovascular devices, which looked to me like a tsunami of new developments. This was probably also due to the innovative five minute presentation format of this very interesting meeting, which sets the ultimate podium for all these new developments, from green ideas to recently launches products – all endovascular and hardly any open surgery, although this was originally a vascular surgery meeting.

Interventional radiologists have always been known as being at the forefront of technological developments. I still remember the cynical T-shirt, once for sale at IR meetings, *“Interventional Radiology: inventing procedures for other specialties since 1973.*” At first glance this role seems now to be taken over by other specialties, like vascular surgery. However, looking more closely, there is also something else going on. While interventional radiologists have mostly been problem driven in inventing new technologies, this new generation of devices often seem to be more a solution for a problem still to be found.

The consequence of this eruption of new devices is that most scientific presentations at endovascular meetings today deal with the safety and very rarely with the clinical efficacy of the new devices. Animal data, first-in-human and small single arm studies supporting these new devices, is numerous. Although this is very inspiring and progress is important, the question remains; how many of these devices will finally make it to the market, and moreover, to our angio-suites with full FDA and/or CE approval? Especially now that the new EU Regulations for medical devices (MDR) and in vitro diagnostic medical devices (IVDR) will be much more stringent in 2020, demanding proof of (clinical?) efficacy.

Also, costs and cost-efficacy in relation to reimbursement is a prominent determiner in daily hospital practise, where evidence-based guidelines are often leading. To give an example; passing an occlusion of the superficial femoral artery can be done either transluminally or, as is mostly the case, subintimally, intentionally or not (Reekers and Bolia 1998). There is no strong data to give a preference to transluminal or subintimal approaches. The technical success has been proven to be more than 80% in experienced hands; only very heavy calcified vessels can sometimes be a problem. This technology, based on experience and skill, is very cheap.

There are now numerous new recanalisation devices available which perform the same as the well-established sub-intimal technique, but should, to be of additional clinical use, foremostly deal with that troublesome 20%. Different older techniques like laser technologies, various forms of atherectomy, and new techniques like shockwave, drills and more also work as recanalisation tools, probably often also via the subintimal route, but they also come at extra costs. They should therefore all be tested head to head with the old and proven, and cheap, revascularisation techniques to establish their added value for money (Reekers 2009; Reekers et al. 1994).

Old, to tackle that misunderstanding, is not the same as redundant, although calling something old certainly brands it as such, like with POBA, Plain (simple, of less value) Old (redundant) Balloon Angioplasty, which is still the basic technique in BTK vessels. Next to all the new devices there is also a fair amount of old technology which seems to be having a third life, sometimes with a little refurbishing by the new owner. Many of these technologies have already been available for more than 25 years, and despite the moderate successes in the past, they are still today often promoted as promising (Reekers 2009; Reekers et al. 1994; van der Lugt et al. 1998; Gussenhoven et al. 1995).

However, patients and indications do not change, and what did not add any value in the past probably does not add any value today, unless you change the indications for treatment. That is where we start to walk on quicksand and where I have my greatest worries about the sustainability of all these new technologies. Fiddling around with indications and study endpoints could very well be the greatest threat for the future and could jeopardize the credibility of endovascular treatments in general.

In the old days, success of treatment for intermittent claudication was always reported as “improvement of walking distance” and success of treatment for critical ischaemia was reported as “prevention of amputation and wound healing”, which are clinical outcomes that matter to the patient. This is no longer the case. Success of treatment is now, almost always, reported by various pseudo or so-called proxy endpoints. TLR (target lesion revascularisation), patency and LLL (late lumen loss) are the parameters for success today instead of the clinical outcomes that really make sense for a patient.

Sadly, none of these proxy endpoints have a proven one to one relation to real clinical endpoints (Reekers 2019).

We have to wait to see how the new European directive for devices will be interpreted, and if efficacy will be measured in meaningful clinical endpoints or if efficacy is also an improvement in TLR and patency. The impact of the new directive for new devices and the re-registration of older technologies is unpredictable today. A golden rule in management is that it is always better to regulate yourself than to wait for others (in this case probably non-medical bureaucrats) to set the rules.

It would be devastating if the new European directives frustrate important technological developments in endovascular treatment. New technologies are needed to move our specialty forward, and I welcome them all. An immediate general agreement among all stakeholders that there should be more focus on meaningful clinical endpoints in reporting new endovascular data is an unavoidable and urgently needed first step for self-regulation. We should, furthermore, also never forget who all this new technology is finally intended to be for. We are doctors and we should treat patients and patient complaints and diseases, rather than only their diseased blood vessels.
